# Reconstruction of H_3_N_2_ influenza virus based virosome in-vitro

**Published:** 2013-06

**Authors:** Asghar Abdoli, Hoorieh Soleimanjahi, Masoumeh Tavassoti Kheiri, Abbas Jamali, Hesam Sohani, Mohsen Abdoli, Hamid Reza Rahmatollahi

**Affiliations:** 1 Department of Virology, Faculty of Medical Science, Tarbiat Modares University, Tehran, Iran; 2 Influenza Research Lab, Pasteur Institute of Iran, Tehran, Iran

**Keywords:** Virosomes, Influenza virus, Reconstruction

## Abstract

**Background and Objectives:**

Virosomes are Virus Like Particles (VLP) assembled in-vitro. Influenza virosomes maintain the cell binding and membrane fusion activity of the wild type virus but are devoid of viral genetic material or internal proteins. Influenza virosomes mimic the natural antigen presentation route of the influenza virus.

**Methods:**

Virosomes were prepared by membrane solubilization and reconstitution. Briefly, the Madine-Darby Canine kidney (MDCK) cell line was cultivated on microcarrier beads inoculated with influenza virus strain A/X-47 (H3N2). The culture medium was harvested and clarified. Subsequently, virus was concentrated and purified by ultrafiltration and ultracentrifugation. The purified viral membrane was dissolved by adding 375 µl of 200 mM 1, 2-dicaproyl-*sn*-glycero-3-phosphocholine (DCPC) in HEPES-buffered saline (HBS). Nucleocapsid was removed by ultracentrifugation. The supernatant consisting of phospholipids and glycoproteins of the influenza virus was reconstituted by removal of DCPC using overnight dialysis against Hank's Buffered Saline (HBS) solution at 4°C. After dialysis, crude virosome preparation was layered over a discontinuous sucrose gradient in order to separate non-incorporated material from the reconstituted virus membranes.

**Results:**

The virosome harvested from the boundary of the two sucrose layers successfully was identified by the Hemagglutination assay and western blotting.

**Conclusion:**

Use of a dialyzable short-chain phospholipid (DCPC) is an efficient procedure for solubilization and reconstitution of influenza virus virosomes and has not caused structural changes in a major envelope glycoprotein (hemagglutinin protein) on the surface of virosome.

## INTRODUCTION

Attachment and fusion of membrane-enveloped viruses with cell receptors is mediated by so called spike glycoproteins on the viral membrane. Almeida *et al*.(1) were the first to report on the construction of lipid vesicles comprising viral spike proteins derived from the influenza virus. Using preformed liposomes, hemagglutinin (HA) and neuraminidase (NA) purified from influenza virus, they succeeded to construct membrane vesicles with spike proteins protruding from the vesicle surface. Observation of these liposomes by electron microscopy revealed that they were very similar to the wild type influenza virus. Consequently, they were named virosomes. In 1987, Stegmann *et al*. described a new procedure for the construction of influenza virosomes by reconstitution of virus-like particles only from viral membrane phospholipids and glycoproteins (2). Afterwards, virosomes have been prepared from numerous enveloped viruses such as influenza virus, Sendai virus, Semliki Forest virus, VSV, Sindbis virus, Epstein-Barr virus, Human Immunodeficiency virus (HIV), Friend Murine Leukaemia virus, HSV and New Castle disease virus (3).

Influenza virosomes are empty influenza virus envelope containing phospholipids and glycoproteins. They are unilamellar vesicles with a mean diameter of 150 nm without internal proteins and genetic materials (4). Virosomes are membranous vesicles that convey viral fusion proteins in their surface. For influenza virosomes this means that influenza HA is anchored in the virosomal surface. For efficient antigen presenting, virosomes should be properly reconstituted such that they can attach and fuse to the same extent as the wild type virus they are derived from (5, 6).

The major viral envelope glycoproteins on the virosomal surface facilitates antigens presentation to the host T and B cells eliciting strong immune responses and stimulation of a balanced Th1/Th2 responses. As well, virosomes have the capacity of fusion with the endosomal membrane, virosome-encapsulated antigens gain access to the MHC class I presentation pathway, consequently inducing cytotoxic T lymphocyte (CTL) responses (7, 8).

The activation of the host immune system mimics the natural infection. In this study influenza A/X-47(H3N2) (a resorted H3N2 (A/Victoria/3/75 (H3N2) and A/Puerto Rico/8/34 (H1N1) virosome was constructed by means of a dialyzable short-chain phospholipid DCPC as a solubilizing agent.

## MATERIALS AND METHODS

### Cell Line

Madin-Darby Canine Kidney (MDCK) cell line with ATCC^®^: CCL-34 was provided by National Cell Bank of Iran, Pasteur Institute of Iran.

### Virus Strain

Influenza virus A/X-47(H3N2) was used in this study kindly provided by Xavier Saelens (University of Ghent, Ghent, Belgium).

### Microcarrier Cell Cultures

The Cytodex 1 microcarriers (Sigma-Aldrich, Sweden) were soaked in PBS pH 7.4 in a 50 ml conical tube for at least 5 h at room temperature. The supernatant was then aspirated out and replaced with fresh PBS. The microcarriers were then sterilized by autoclaving at 120°C for 15 min. PBS was removed and exchanged with growth Dulbecco's Modified Eagle's Medium (DMEM, Gibco) containing 20% filtered fetal bovine serum (FBS, Gibco) and 150×10^6^ cells/L was added and pumped into the spinner flask (Cellspin Integra Biosciences) at pH 7.4 at 37°C and kept shaking at 55 rpm (9).

### Virus inoculation

Virus quantified by Cell Culture Infective Dose 50% (CCID_50_) assay. Cell culture growth medium was replaced with DMEM containing antibiotics (100 IU /mL penicillin and 100 µg /ml streptomycin) without serum, with 2 µg /ml L-1-tosylamido-2-phenylethyl chloromethyl ketone (TPCK)-treated trypsin (sigma). Cell were infected with 0.1 multiplicity of infection in spinner flask and harvested after 72 h (9, 10).

### Clarification and primary concentration

Cell debris and cytodex 1 were removed by twice centrifugation at 6000 rpm for 10 min. Titration of the viruses in the supernatant was carried out by hemagglutination assay and dilutions equal and above 1: 1024 was chosen as the optimal titer for further work. The clarified supernatant run to Tangential Flow Filtration (TFF) ultra-filtration set (Millipore, USA) for primary concentration with a 100 KD cut off filter (11).

### Concentration and purification

Discontinuous sucrose gradient Ultracentrifigation (10%/60% w/v) was applied for 1.5 h (100.000 x g at 4°C at Optima XL-90 Beckman rotor Ti-90, USA) for further concentration and purification.

The final purified viruses were harvested from the boundary of the two sucrose layers (10%/60% w/v), and dialyzed against HBS at 4°C for overnight to eliminate the residual sucrose. Subsequently, dialyzed influenza viruses were sedimented by ultracentrifugation (100.000 x g for 1 h at 4°C) and the 5 mg of pure virus pellet was suspended in 375 µl of Hank's Buffer (11).

### Solubilization of the viral membrane and ribonucleoprotein removal

Virus membrane was solubilized by addition of 375µl 200 mM 1, 2-dicaproyl-sn-glycero-3-phosphocholine (DCPC) in HBS. The viral Ribonucleoproteins (RNPs) were removed by ultracentrifugation (85.000 x g for 30 min at 4°C) (12, 13).

### Reconstitution of virus membrane

After removing of RNP, the supernatant containing the viral glycoproteins and lipids were recovered. Rebuilding of the virus membrane was accomplished by removal of DCPC by means of dialysis. The reconstituted virus envelope named prepared crude virosome.

### Purified virosome preparation

In order to separate non-incorporated material from the reconstituted virus membranes the crude virosome preparation were purified by discontinuous sucrose gradient (10%/60% w/v sucrose in HBS) and centrifuged for 1.5 h (100.000 x g at 4°C). The purified virosome were harvested from the boundary of the two sucrose layer and dialyzed against HBS overnight at 4°C to remove the sucrose. The protein content of the virosomes analyzed by micro Lowry assay (3).

### Confirmatory assay for virosome structure: Hemagglutination Assay (HA)

HA was used in order to show whether the reconstituted virosome is able to agglutinate the red blood cells via its surface glycoproteins 50 µl of the purified virosome were diluted in two-fold dilution with PBS and 50µl of a 0.5% suspension of chicken red blood cells were added to each dilution in a U-shaped microtitre plate. After a gentle agitation, the plates were left undisturbed for 30 min at room temperature. The last dilution showing complete hemagglutination was taken as the end point and was expressed as hemagglutination unit (HAU) per test volume (14, 15).

### Sodium Dodecyl Sulfate Polyacrylamide Gel Electrophoresis (SDS-PAGE) and Western Blotting

The purefied H3N2 virosome were loaded on 12% polyacrylamide gel to perform SDS-PAGE and transferred onto nitrocellulose membrane (0.45 µm, S&S Bioscience GmbH, Whatman group, D assel, Germany) by semidry electro-transfer (Applex, 016932). The blotting was carried out in transfer buffer (10% methanol), 24 mM Tris (Sigma-Aldrich), and 194 mM glycine (Sigma-Aldrich), pH 8, at 10 V for 30 min. For blocking Nonspecific binding the membrane incubated in blocking solution containing 2.5% bovine serum albumin (Roche, Mannheim, Germany) in phosphate-buffered saline for 1.5 h at room temperature. The blotted HA protein was reacted with mouse primary monoclonal antibody (Abcam, USA) specific to HA of H3N2 virus. Mouse IgG secondry antibody (Sinobiological) conjugated with HRP was applied as secondary antibody. The TMB substrate solution (Sigma-Aldrich) was added to see reacted protein bands (16).

### Infectivity and Toxicity assay of virosome

In order to evaluate the toxicity and infectivity of the constructed virusome, 5, 10 and 15µg of them transfected to Vero and MDCK cells. The cells monitored for 72 hours post infection.

## RESULTS

**Fig. 1:**
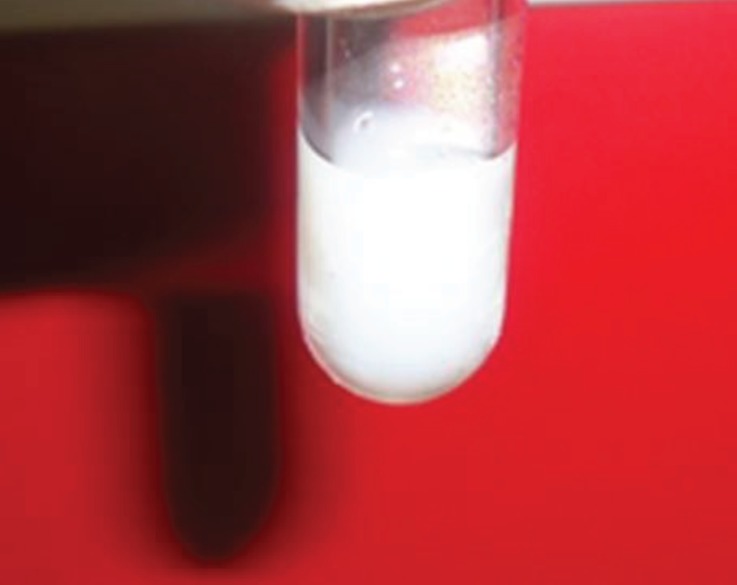
H_3_N_2_ virus after concentration and purification in HBS.

The crude viruses were concentrated approximately 50 times post concentration and purification by ultrafiltration and ultracentrifugation (Fig. 1).

### Protein amount determination

Total proteins of reconstituted influenza virosome as well as the whole virus before and after concentration and purification were determined by Lowry assay. We have shown the purified virosomes contained roughly 10% of the total viral proteins (Table 1).

**Table 1:** Protein concentration and Hemagglutination titer of the virus and the virosome.

	Cell culture of the virus	Concentrated and purified virus	The virosome
**Protein concentration**	100 µg/ml	5 mg/ml	500 µg/ml
**Hemagglutination titer**	10240 HA/ml	655360 HA/ml	81920 HA/ml

**Fig. 2:**
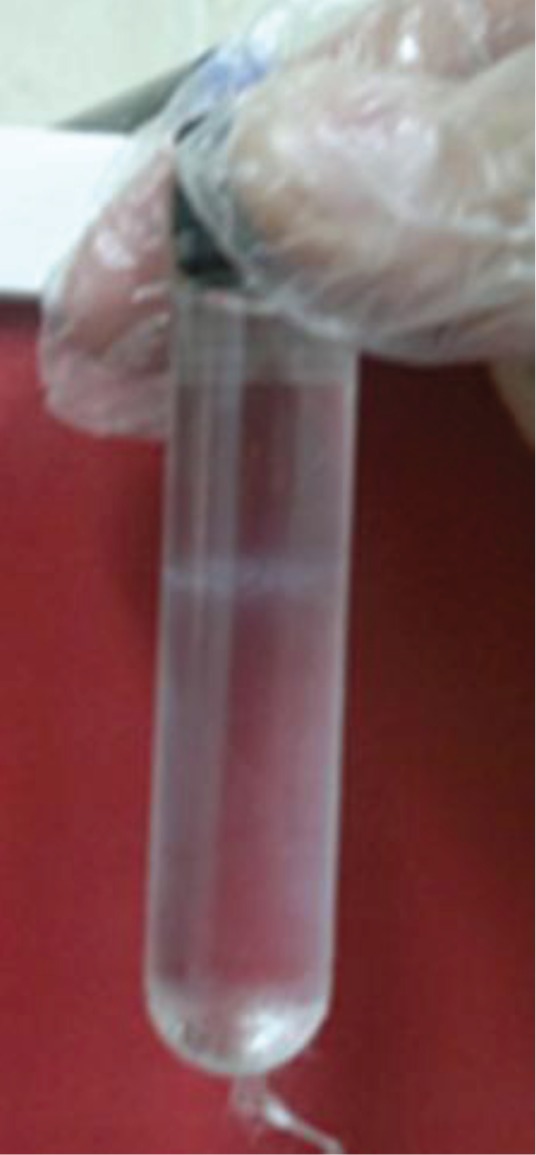
The H3N2 influenza based virosome: Viral envelopes were resolved using DCPC, genetic materials and internal proteins were removed and the virosome was prepared by dialysis. The virosome was purified by sucrose gradient and ultracentrifugation.

Influenza virus buoyant density in sucrose is 1.19-1.21 g/ml; therefore, we used 10% and 60% of step gradient sucrose for purification of influenza virus as have been shown in Fig. 2.

**Fig. 3:**
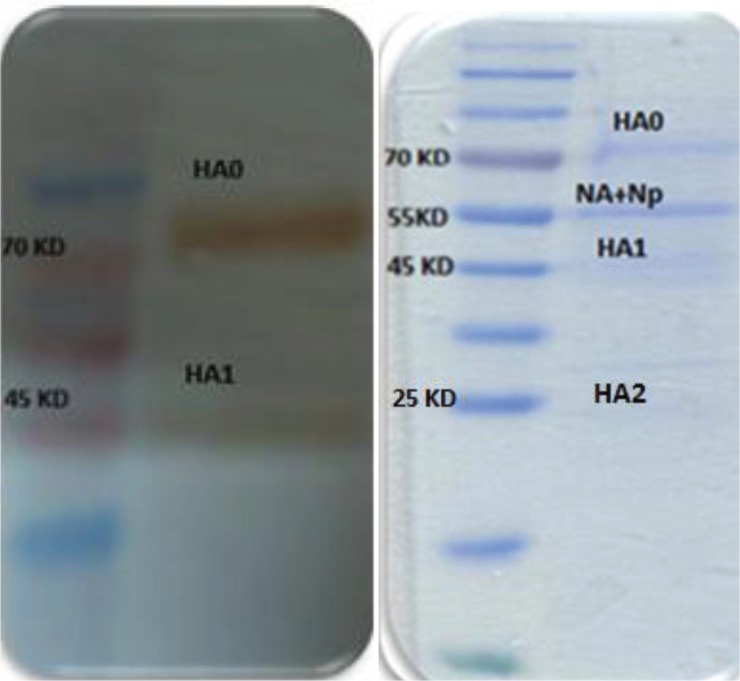
SDS-PAGE and Western Blot analysis of the purified virosomes: The constructed H3N2 virosome (10 g) loaded and proteins were separated on 12% polyacrylamide gel and stained with Coomassie blue. Influenza A virus Hemagglutinin H3 monoclonal antibody was used for Western blotting.

Two main virus glycoproteins are inserted into the reconstituted virosome, haemagglutinin (HA) and neuraminidase (NA) (Fig. 3). The receptor binding feature of incorporated hemagglutinin in virosomes was determined by HA assay. Influenza virosomes demonstrated 81920 HA/ml agglutination activity (Table 1).

The target of neutralizing antibodies that protect against influenza virus infection is the haemagglutinin (HA). The antibody binding activity of HA evaluated by Western blotting.

The complete haemagglutinin (HA0 = 75 kD) is composed of two separate subunits, termed HA1 (55 kD) and HA2 (20 kD), joined together by a disulphide bond. They are separated during SDS-PAGE because of reducing agent, so three bands could be seen in SDS-PAGE (HA0, HA1, HA2) and two bands (HA0, HA1) in Western blotting as it is shown in Fig. 3 (17).

### Infectivity assay of virosome

Infectivity of virosome was monitored 72 h post transfection. There was no visible cytopathic effect on cell lines in comparison with control cells.

## DISCUSSION

Current commercial influenza vaccines are whole inactivated virus, attenuated virus, split virus, or subunit antigen. Inactivated influenza vaccines create great deals of protection against influenza but these vaccines are not ideal for older adults because of their decreasing immunity and other factors affecting immunogenicity (18). Virosomes expose another novel influenza vaccine strategy, stimulating the immune system as in a natural infection. Studies have suggested that the virosomal adjuvanted influenza vaccine is immunogenic and safe in different population groups including children, elderly and immunocompromised individuals as compared to conventional vaccines (19). Gluck *et al*. compared the virosomal influenza vaccine with an inactivated whole vaccine and a subunit vaccine and revealed to prompt significantly higher seroconversion rates (83%, 79% and 67% for A/H1N1, A/H2N2 and B, respectively) than the comparators for all three strains (20, 21).

Influenza virosomes are capable of performing both requirements as a candidate vaccine. Firstly, they act as an antigen presenting system to stimulate both the humeral and cellular host immune system. Secondly, virosomes have the opportunity to incorporate lipophilic or amphiphilic antigens to induce the antibody response against the viral haemagglutinin (22).

The influenza virosomal vaccine (Inflexal VR, Crucell, Switzerland) has been used broadly in several European countries since 1997 and has presented good results in terms of tolerability and safety in elderly, children and immonocompromiseds. It is the first influenza vaccine licensed for all age groups due to its biocompatibility and purity. Clinical studies have proven that intramuscular administration of virosomes enhance haemagglutination–inhibition (HI) titers in human, akin to those induced by conventional whole virus or subunit vaccines (23).

Licensed adjuvants can be categorized into 3 main classes: mineral salts (aluminium hydroxide, alum), oil emulsions (MF59, AS03 and AF03) and particulate delivery vehicles (virosomes, AS04). In addition to Inflexal^®^ V, influenza virosomes as an adjuvant have been efficiently used in the development of the Epaxal^®^, which has been on the market since 1994 for vaccination of Hepatitis A virus (24). The influenza virosome as an adjuvant has been recently used for formulation of Malaria and leishmania vaccines (25, 26). Application of influenza virosome as a delivery system for protein\peptide, siRNA and nucleic acids was studied previously (22, 27).

Taken together, in this research we produced and characterized the influenza A/X-47(H3N2) virosome by means of a dialyzable short-chain phospholipid DCPC as a detergent. Since the virosome has a virus like structure having HA and NA glycoproteins on the surface, it could mimic the hemagglutination capability on the virus. Hemagglutination assay confirmed that the glycoprotein HA was truly incorporated into virosomes retaining its biological activity. The study of the vaccine to evaluate capability of the virosome to induce immune responses against influenza virus in animal models is undergoing.
